# Primary retroperitoneal mucinous cystic tumour of borderline malignancy mimicking kidney duplicate: cases report and literature review

**DOI:** 10.1186/s12894-023-01191-z

**Published:** 2023-03-05

**Authors:** Junlong Zhang, Qinsong Zeng, Jihui Kang, Junxing Chen, Guiyuan Luo, Yueyou Liang

**Affiliations:** 1 Department of Urology, The First Affiliated Hospital of Sun Yat-Sen University, No. 58 Zhongshan Er Road, Guangzhou, 510080 China; 2 Department of Pathology, The First Affiliated Hospital of Sun Yat-Sen University, Guangzhou, China; 3 Department of Anesthesiology, The First Affiliated Hospital of Sun Yat-Sen University, No. 58 Zhongshan Er Road, Guangzhou, 510080 China

**Keywords:** Duplex kidney, Primary retroperitoneal mucinous cystadenoma, Surgical approach

## Abstract

**Background:**

Primary retroperitoneal mucinous cystic tumours with borderline malignancy (PRMC-BM) are rare and difficult to diagnose preoperatively. We are the first to report two cases of PRMC-BM which mimic a duplex kidney and evaluate the outcomes of different surgical procedures.

**Case presentation:**

We describe two cases of retroperitoneal cystic tumours. Both were diagnosed with duplex kidney with hydronephrosis on computed tomography scan. The first patient underwent robot-assisted laparoscopic surgery and was found to have a retroperitoneal cystic tumour. The other patient underwent an ultrasound-guided puncture before surgery and was diagnosed with retroperitoneal lymphangioma. Retroperitoneal cystectomy was performed using an open transperitoneal procedure. The final pathologic diagnosis in both cases implies PRMC-BM. The open surgical approach was associated with a shorter operation time, less intraoperative blood loss, and protected cyst wall integrity by comparing the different surgical approaches. During follow-up, the patient in the first case had tumour recurrence six months post-surgery, and the other patient was healthy without recurrence or metastasis 12 months post-surgery.

**Conclusions:**

Primary retroperitoneal mucinous cystic tumours with borderline malignancy can be enclosed within the kidney and misdiagnosed as other cystic diseases of the urinary system. Thus, an open surgical approach may be more suitable for this type of tumour.

## Background

Primary retroperitoneal mucinous cystic tumour with borderline malignancy (PRMC-BM) is an extremely rare disease [1]. Only 23 cases have been reported, and only one exhibited metastasis [1, 2]. No PRMC-BM mimicking duplicated kidneys have been reported in the literature. In this report, we present two cases of PRMC-BM mimicking duplicated kidneys. We also provide a literature review and discuss the outcomes of different surgical procedures.

## Case presentation

### Case #1

**Fig. 1:**
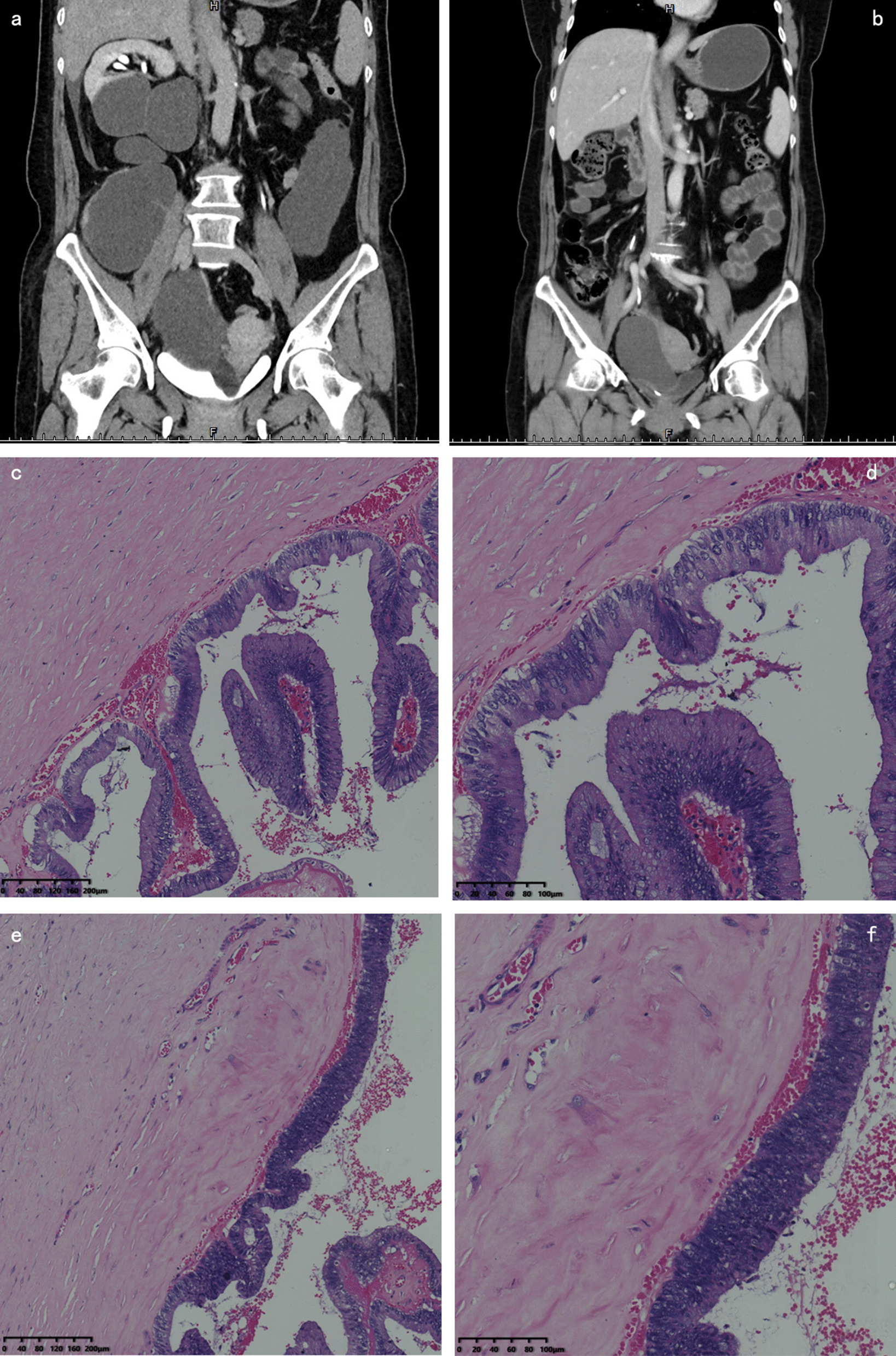
**a** Coronal computed tomography (CT) image of the retroperitoneal cystic tumour. **b** Coronal CT image of the tumour recurrence after six months of surgery. **c**, **d** Low-grade intraepithelial neoplasia in the cyst wall. **e**, **f** Focal high-grade intraepithelial neoplasia in the cyst wall. (haematoxylin–eosin stain; **c**, **e**: magnification: × 100; **d**, **f**: magnification: × 200)

A 56-year-old woman presented with painful urination and discomfort in the right waist for one year. Examination revealed irregular cystic masses (diameter, 25 cm) in the right abdomen. Abdominal computed tomography (CT) revealed irregular low-density lesions measuring approximately 28 × 22 × 8 cm in the right ureter area, beginning from the lower pole of the right kidney to the bladder neck. Contrast-enhanced CT showed obvious enhancement of the cyst wall, but the borderline of the lesion and the lower part of the right ureter were unclear during the renal excretion stage (Fig. 1a). The preoperative diagnosis of this patient was a duplicate right-sided kidney malformation with renal ureteral dilatation.

A partial nephrectomy for a duplex kidney was scheduled using a robot-assisted laparoscopic approach. The trocars were successfully established. After the lateral peritoneum was opened, we found an independent cystic mass close to the right kidney, rather than a duplicated kidney malformation. The tumour cyst wall was then opened and a cream-like liquid was observed. Another cystic mass was found under the tumour. Both lesions were removed.

The operation time was 470 min, and intraoperative blood loss was approximately 600 mL. The patient recovered well and was discharged four days postoperatively without any postoperative complications. The final pathological diagnosis was PRMC-BM (Fig. 1c–f). During follow-up, the patient experienced tumour recurrence six months after the operation (Fig. 1b).

### Case #2

**Fig. 2:**
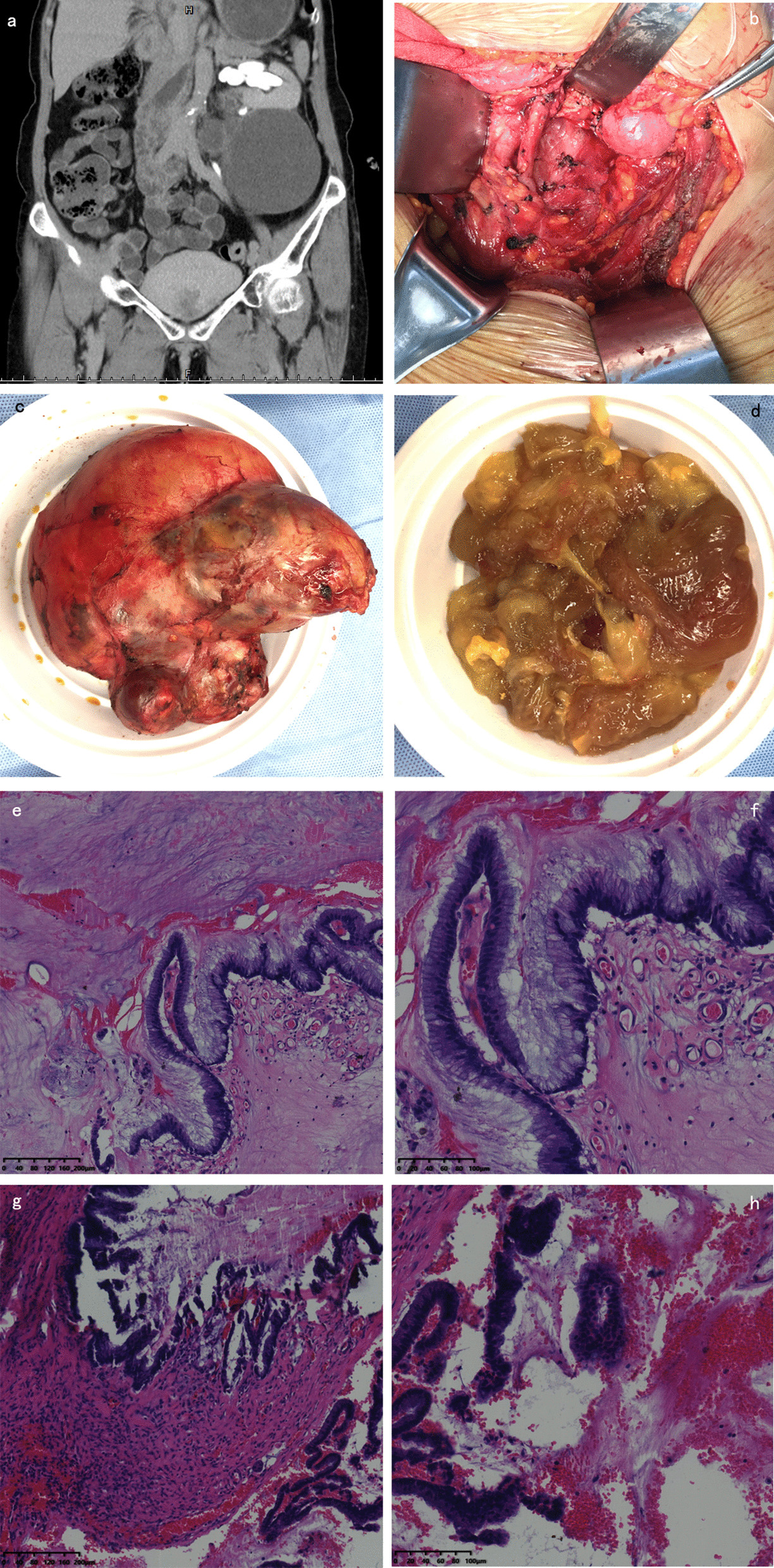
**a** Coronal computed tomography image of the retroperitoneal cystic tumour. **b** Double ureter showed in the retroperitoneum after the tumour removal. **c** The cystic mass. **d** The fluid content of the tumour. **e**, **f** Low-grade intraepithelial neoplasia in the cyst wall. **g**, **h** Focal high-grade intraepithelial neoplasia in the cyst wall. (haematoxylin–eosin stain; **e**, **g**: magnification: × 100; **f**, **h**: magnification: × 200)

A 68-year-old woman was admitted to our hospital with a mass in the lower left retroperitoneum. A physical examination revealed a local bulge in the left lower abdomen, and a palpable mass approximately 15 × 10 cm in size. The mass was tough and could be moved without tenderness. Abdominal contrast-enhanced CT revealed a cystic mass with the size of 15 × 11 × 9 cm below the left kidney. The borderline between the cystic mass and the left ureter was unclear. The left kidney showed a double renal pelvis and ureter accompanied by hydronephrosis and dilatation of the ureter. There was no obvious enhancement of the cyst wall or cyst (Fig. 2a). It was difficult to differentiate the cyst from a duplicated kidney with hydronephrosis. Ultrasound-guided puncture of the cyst was performed, and the cyst fluid was viscous but not urine. Thus, the preoperative diagnosis of this patient was retroperitoneal lymphangioma.

Retroperitoneal mass resection was performed using an open approach. Before tumour resection, a double J was placed in the left ureter. A transverse incision of approximately 15 cm in length was made on the left abdomen. After exploring the peritoneum, a large cystic mass was observed below the lower pole of the kidney. The mass surrounded the ureter and was underneath the renal hilum. The mass was completely removed without secondary injury to the cystic wall. The duplicated ureters were intact (Fig. 2b). The cystic mass was then dissected and showed viscous fluid and pale yellow jelly (Fig. 2c and d).

The operation time was 210 min, and intraoperative blood loss was approximately 30 mL. The patient recovered well and was discharged six days postoperatively without any complications. The final diagnosis was PRMC-BM (Fig. 2e–h). No additional therapy was administered, and the patient was healthy without recurrence or metastasis 12 months after the surgery.

## Discussion and conclusions

Mucinous cystadenomas are common in the ovaries, pancreas, and appendices. It is thought to be a PRMC when located in the retroperitoneum with a normal visceral organ[3]. According to its pathology, there are three types of PRMC: mucinous cystadenoma, mucinous cystic tumour of borderline malignancy, and mucinous cystadenocarcinoma[4]. Using Medline, we performed a literature review since 1966, and only 23 PRMC-BM cases have been reported (Table 1) [1, 2, 4–20].

**Table 1:** Previously published cases of primary retroperitoneal mucinous cystic tumours with borderline malignancy

Study	Age (years)	Symptom	Image features	Location	Preoperative diagnosis	Therapy	Outcome
Banerjee et al	38	Abdominal pain and distension	Mul cyst	Lt. Lower Abdomen	ND	TR and DC, Lt. Ft and ov	Lymph metastatic, 48 mo
	47	‘flu-like’ symptoms	ND	Lt adrenal	adrenal tumour	TR and spleen and Lt. adrenal	ND
Motoyama et al	42	ND	ND	ND	ND	ND	NED
	63	ND	ND	ND	ND	ND	ND
Pearl et al	33	Abdominal swelling, pain	Un cyst	Lt. Flank	ND	LR in fragments	NED,10 mo
Papadogiannakis et al	33	Abdominal mass	ND	DC	Mes.cyst	TR	NED,12 mo
Chen et al	48	Abdominal fullness	ND	AC	Mes.cyst	LR	NED,12 mo
Gutsu et al	41	Flank pain,fullness	Un cyst	Below the Rt. kidney	RP cyst	TR	NED,18mo
Song et al	31	Abdominal distension in the Rt. lower quadrant	Mul cyst	the Rt. RP	RP cyst	TR	ND
Matsubara et al	36	Abdominal distension	Mul cyst nodule	Rt RP space	Rt. ov cyst	TR	NED,6 mo
Bakker et al	45	Abdominal pain	nodule	Near the pan	papillary adenocarcinoma	TR	NED,12 mo
Cottrill et al	22	Abdominal pain and distension	ND	Superior to the uterus	Lt. ov cyst	TR	NED,24 mo
Bifulco et al	35	Pelvic pain	Un cyst	Between pan and GB	RP cyst	TR	NED,24 mo
Roma et al	25	Kidney mass	Un cyst with papilla	ND	ND	ND	NED, 148 mo
	43	Pelvic pain	Un cyst with papilla	ND	ND	ND	NED,1 mo
	48	Enlarged mass	Mul cyst	ND	ND	ND	NED, 34 mo
Benkirane et al	44	Mass, Rt. Abdominal pain	Mul cyst	pre-aortic and inter aor-tocave areas	ND	TR	NED,12 mo
Falidas et al	37	Rt. lateral abdomen pain	Mul cyst	the inferior pole of the Rt. kidney to the iliac crest	ND	TR	NED,12 mo
Mattei et al	32	ND	ND	ND	ND	LR	ND
Haeri et al	26	Abdominal distention and pain	ND	Lt. lower quadrant of the abdomen	ND	TR	ND
Manrai et al	65	Abdominal distension	Mul cyst	Pelvic	Lt. ov cyst	TR	NED,12 mo
Vargas et al	68	Abdominal mass	Mul cyst	Rt. abdomen	potential malignant PR tumour	TR	ND
Mariana et al	62	Abdominal pain	Un cyst	Rt flank close relate with the cecum and caecal appendix	ND	LR and ileocecal resection	NED,18 mo
Present cases	56	Painful urination and Flank pain	Mul cyst Enh	Rt. kidney	Kidney Duplicate	LR	Tumor recurrence,6 mo
	68	Abdominal mass	Mul cyst no Enh	Lt. kidney	RP cyst	TR	NED,12 mo

AC, Ascending colon; DC, Descending colon; Enh, enhancement; Ft, Fallopian tube; GB, gallbladder; LR, laparoscopic resection;

Lt., left; Mes, mesenteric; Mul,Multilocular; ND, not described; NED, no evidence of disease; ov, ovarian; pan,pancreas;

PR, retroperitoneal; Rt., right; TR, tumor resection; Un, Unilocular

Preoperative diagnosis of PRMC is very difficult. Imaging is helpful in the detection of retroperitoneal cystic masses, but it is difficult to differentiate PRMC from a variety of retroperitoneal cystic diseases because of the lack of typical imaging features[3]. In the 23 cases reported in the literature, the age of the patients ranged from 22 to 68 years. Most patients presented with local swelling, pain, and self-examination of the mass, similar to our two cases. As shown in Table 1, most cysts were located in the pelvis or near the colon. Although it is close to the kidney, the cystic mass often appears as squeezing the kidney and is easily differentiated from hydronephrosis. Here, we report the first case of PRMC-BM mimicking kidney duplication. In our first case, a huge retroperitoneal cystic mass located close to the kidney was misdiagnosed as malformation of a duplicate kidney, and a robot-assisted laparoscopic partial nephrectomy for a duplex kidney was scheduled. We found that ultrasound-guided puncture of the cyst may be useful in the differential diagnosis when a duplicate kidney is suspected. In our second case, the fluid in the cyst was very viscous, and hydronephrosis was excluded. Percutaneous cyst puncture of the cystic mass and cytological examination of cystic fluid were performed in some cases[6, 21], similar to our second case, which did not seem to increase the risk of tumour implantation and recurrence.

The occurrence of PRMC is most commonly seen in female patients, but its histogenesis remains unclear. The main theories are as follows: seeding of ectopic ovarian tissue[22], monodermal variant of teratomas, enterogenic duplication of cysts, and coelomic metaplasia[23]. Retroperitoneal cystectomy is considered an effective treatment for PRMC. Both open and laparoscopic surgical approaches have been reported for the treatment of PRMC-BM, and the outcome is generally favourable [4]. Surgeons prefer the open approach. The laparoscopic surgical approach is thought to have the advantage of being minimally invasive[21], and cyst aspiration and fragment removal do not affect prognosis[6]. We recommend an open surgical approach for this type of tumour. First, the cystic mass is often large and needs to be aspirated when using a laparoscopic surgical approach, which carries the risk of tumour implantation. In our first case, the patient experienced tumour recurrence six months after cystectomy using a robot-assisted laparoscopic approach, which may be related to the opening of the cyst. Moreover, the cystic fluid could be viscous and difficult to aspirate which may greatly increase the difficulty of the operation. Second, the ureter encapsulated by the mass can be touched by open surgery and, thus, can be safely retained. In our second case, the cystic mass was large and half-encapsulated in the kidneys and ureters. A double J was placed in the left ureter before tumour removal. We found that it was very easy to find the location of the ureter and thus avoid ureteral injury. Third, the open surgical approach had a shorter operation time and less intraoperative bleeding, suggesting that the open surgical approach is safer.

Furthermore, PRMC-BM is a potential malignant type of PRMC. However, we reviewed other reports and found that the recurrence rate in such cases was very small. We tried to analyse the imaging features of the cystic lesions, multilocular, enhanced, and solid nodules but failed to reveal any regularity in the recurrence rate. Chemotherapy is recommended for mucinous cystadenocarcinoma[1]. However, in PRMC-BM, only close follow-up is required.

In conclusion, PRMC-BM is a rare disease that may mimic urinary tract-related diseases, and urologists need to be aware of it. Ultrasound-guided puncture of the cyst may be useful in differential diagnosis before surgery. Since cystic masses can be malignant, careful protection of the cyst wall and prevention of cyst rupture can reduce the risk of tumour recurrence, and an open surgical approach may be more appropriate.

## Data Availability

The data and materials used in this study are available from the corresponding author upon request. All authors have read the paper and agree that it can be published elsewhere.

## Abbreviations

PRMC: Primary retroperitoneal mucinous cystadenoma
PRMC-BM: Primary retroperitoneal mucinous cystic tumour with borderline malignancy
